# My Cosmos: development, clinical protocol, and preliminary usability of a gamified transdiagnostic digital CBT platform

**DOI:** 10.3389/fpsyt.2026.1792957

**Published:** 2026-04-10

**Authors:** Sasha Del Vecchio, Tommaso Barlattani, Antonino Carcione, Francesca Pacitti, Marco Recchioni

**Affiliations:** 1 Department of Biotechnological and Applied Clinical Sciences (DISCAB), University of L’Aquila, L’Aquila, Italy; 2 Department of Human Sciences, Guglielmo Marconi University, Rome, Italy; 3 Third Centre of Cognitive Psychotherapy, Scuola Italiana di Cognitivismo Clinico (SICC), Rome, Italy; 4 Department of Life, Health and Environmental Sciences, University of L’Aquila, L’Aquila, Italy

**Keywords:** blended care, digital cognitive behavioural therapy, digital mental health, gamification, transdiagnostic intervention, usability and acceptability

## Abstract

**Background:**

Digital cognitive–behavioural therapy (dCBT) has been shown to be effective for anxiety and depression, yet sustaining engagement remains a key challenge that limits real-world impact. Gamification and extended reality (XR) elements may support motivation and skill transfer, but few interventions integrate these features within a clinician-compatible, clinically grounded framework. My Cosmos is an internet-delivered, mobile-first transdiagnostic dCBT programme that embeds first-wave CBT alongside selected second- and third-wave approaches within a game-inspired structure designed to support adherence and self-regulation.

**Methods:**

My Cosmos was developed through evidence mapping, co-design with potential users and mental health professionals, iterative prototyping, and implementation of GDPR-compliant pseudonymised data flows. The programme assigns users to Anxiety, Depression, or Mixed tracks based on baseline PHQ-9, GAD-7, and SF-36 scores. Each track follows a six-module CBT pathway enriched with gamified Rehabilitation Objects and supported by a secure clinician web dashboard to enable blended care. Preliminary usability and acceptance were assessed through participatory workshops with university students (N = 124) and mental health professionals (N = 19), using standardised questionnaires (SUS, MAUQ, UTAUT2) and inductive qualitative analysis of open-ended feedback.

**Results:**

Help-seekers described My Cosmos as intuitive, engaging, and emotionally supportive, emphasising its usefulness for self-regulation and structured reflection. Mental health professionals reported more moderate but overall positive evaluations, recognising the platform’s value for supporting homework, monitoring progress, and enhancing continuity between sessions, while highlighting the importance of clear onboarding, transparent communication about data protection, and flexible clinical integration. Both groups converged on a blended-care positioning in which the programme complements, rather than replaces, the therapeutic relationship.

**Conclusions:**

This paper presents the development, clinical protocol, and early-stage usability evaluation of My Cosmos, a gamified, modular, transdiagnostic dCBT programme implemented within a secure digital environment. Findings inform ongoing optimisation and support progression toward beta/feasibility testing and a planned randomised controlled trial to evaluate clinical effectiveness, engagement, and health-economic outcomes.

## 1 Introduction

Digital transformation is reshaping health care delivery, and mental health is no exception. Digital Health Technologies (DHT) include tools that support assessment, monitoring, and treatment decisions and, increasingly, can also deliver therapeutic content directly to patients (1). When the software itself constitutes the active, evidence-based intervention—implemented as a structured therapeutic programme, evaluated in clinical studies, and intended for use within clinical pathways—it is commonly referred to as a digital therapeutic (DTx) (2). Within mental health, one of the most established DTx modalities is digital cognitive–behavioural therapy (dCBT), often implemented as internet-delivered CBT that can be embedded within stepped-care and blended-care models rather than positioned as a standalone substitute for clinician-led treatment (3–6).

Regulatory, commissioning, and reimbursement pathways are evolving alongside the evidence base. In Europe, national routes such as Germany’s Digital Health Applications programme (DiGA; in German: Digitale Gesundheitsanwendungen) and emerging assessment and reimbursement initiatives in other countries have expanded the availability of certified digital medical devices and therapeutics, while the United States has seen a growing number of prescription digital therapeutics evaluated and authorised through the FDA medical-device framework (7, 8). In parallel, the National Institute for Health and Care Excellence (NICE) has issued an evidence standards framework for digital health technologies and has published early value assessment guidance on digitally enabled therapies for adults with depression and anxiety disorders, recommending their use within appropriate governance and service models (9, 10). Together, these developments underline a key implementation insight: outcomes depend not only on clinical content, but also on onboarding, safety governance, monitoring, and integration into real-world workflows.

A substantial evidence base supports dCBT for anxiety and depression. Meta-analytic syntheses suggest that therapist-supported internet-delivered CBT can achieve outcomes comparable to face-to-face CBT, whereas fully self-guided interventions tend to show smaller effects and higher attrition (3, 5, 11). For adult depression, an individual participant data (IPD) network meta-analysis found both guided and unguided iCBT to outperform control conditions, with guided iCBT showing a small additional post-treatment benefit over unguided iCBT (mean difference in post-treatment PHQ-9 scores −0.8, 95% CI −1.4 to −0.2) and evidence that baseline symptom severity and employment status contribute to outcomes (4). Evidence from randomised trials also suggests that some smartphone app interventions can reduce depressive symptoms compared with control conditions (g = 0.38, 95% CI 0.24–0.52), although these estimates apply to apps evaluated in controlled trials and should not be generalised to the wider commercial marketplace, where many products lack robust evidence (12). Beyond standard CBT protocols, digital programmes increasingly incorporate transdiagnostic and third-wave elements, including online mindfulness-based interventions, internet-delivered Acceptance and Commitment Therapy (ACT), and metacognitive techniques, thereby targeting processes such as rumination, worry, and experiential avoidance (13–16).

Despite clinical promise, engagement and adherence remain major barriers to sustained benefit at scale. The “law of attrition” describes the characteristic drop-off seen in eHealth interventions, particularly when support is minimal or perceived relevance diminishes (17). Reviews suggest that adherence is shaped by usability and persuasive design (e.g., reminders, tailoring, frictionless navigation), the availability and intensity of human support, and the perceived therapeutic alliance with the programme (18–21).

Two complementary innovation directions are particularly relevant for addressing engagement and supporting skills transfer. First, gamification and serious-game elements may strengthen motivation and experiential learning when tightly coupled to therapeutic objectives rather than added as superficial reward layers (22–24). Second, rule-based and adaptive tailoring—ranging from baseline triage and mastery-based progression to more dynamic sequencing—may reduce mismatch between user needs and therapeutic content (25–27). Immersive technologies may further support in-vivo practice in selected CBT applications: virtual reality (VR) has been studied primarily as an adjunct for exposure-based interventions, while augmented reality (AR) may enhance ecological validity by delivering brief, context-aware tasks in everyday environments (28, 29).

Within gamified and serious-game approaches, SPARX—a CBT-based fantasy game for adolescents with depression—is an early exemplar and has been widely cited in the field; subsequent reviews and protocols continue to highlight its clinical potential while noting engagement and adherence as key implementation drivers (30–32). Other examples include SuperBetter, which embeds CBT- and positive psychology–informed strategies within a quest-based framework and has shown improvements in depressive symptoms in controlled research (33). Overall, review evidence suggests that gamified mental health interventions can yield small-to-moderate benefits, although effects vary across populations (e.g., adolescents vs adults; clinical vs subclinical samples), delivery models (self-guided vs guided/blended), and the specific mechanics used (e.g., narrative quests, points/badges, feedback loops, social features) (26, 34, 35).

My Cosmos was developed in this space as an internet-delivered, mobile-first, cross-platform transdiagnostic dCBT programme for adults with mild-to-moderate anxiety and/or depressive symptoms. The programme assigns users to Anxiety, Depression, or Mixed tracks through baseline psychometric screening, delivers protocolised modular pathways, and embeds first-wave CBT as the core therapeutic engine while incorporating selected third-wave and metacognitive micro-skills. Therapeutic techniques are learned and rehearsed through a game-inspired, role-based format in which help-seekers adopt the role of a psychotherapist guiding narrative-driven fictional characters through common emotional and behavioural difficulties, an approach intended to support reflective processing and skills generalisation (36). The platform includes a structured triage and safety gateway, supports clinician oversight via a secure dashboard to enable blended care, and implements privacy-by-design through GDPR-compliant pseudonymised data flows. XR is operationalised in a lightweight way through optional smartphone-based AR micro-tasks (e.g., behavioural-activation “treasure hunts”) designed to promote real-world practice without requiring dedicated hardware.

The present paper focuses on the development and preliminary usability evaluation of My Cosmos. Specifically, we address the following questions: (1) How was My Cosmos developed, including evidence mapping, co-design, and iterative prototyping decisions? (2) What are the key features of the current clinical protocol and safety/governance approach (triage, module sequencing, mastery-based progression, and blended-care support)? (3) How do potential end-users and mental health professionals evaluate early usability and acceptability, and what actionable design requirements emerge from their feedback? Answering these questions provides design and implementation insights to guide ongoing optimisation and subsequent evaluation phases (including feasibility and effectiveness studies).

## 2 Materials and methods

### 2.1 State of the art analysis

Before drafting wireframes or therapeutic content for My Cosmos, we conducted a two-step evidence mapping and benchmark analysis to inform both therapeutic architecture and interaction design decisions.

First, structured searches were performed in PubMed (MEDLINE) and Scopus (accessed 15 June 2025) to identify digital interventions targeting anxiety, depression, or transdiagnostic emotional problems that were supported by at least one peer-reviewed evaluation study (e.g., clinical outcomes, feasibility, or implementation studies). Searches combined condition-related terms (e.g., anxiety, depression, transdiagnostic, emotion regulation) with delivery and treatment terms (e.g., digital, internet-based, mobile app, digital intervention, CBT, cognitive behavioural therapy). Screening was conducted at title/abstract level, with full-text inspection where needed to confirm eligibility and the presence of evaluative evidence.

Second, the Apple App Store and Google Play Store were examined (accessed 15 June 2025) to benchmark visual style and interaction design patterns among widely used mental health and wellbeing applications. App-store candidates were identified through category browsing (e.g., Health & Fitness/Medical, where applicable) and keyword searches (e.g., anxiety, depression, CBT, mindfulness, mood tracker). These commercially popular apps were included to benchmark real-world UI/UX conventions and engagement patterns and were not treated as evidence-based interventions unless peer-reviewed outcome evidence was identified via the database searches.

Based on the combined outputs of database screening and app-store benchmarking, a heterogeneous set of applications was selected for analysis, encompassing mindfulness-oriented wellness apps, therapist-supported and self-guided CBT programmes, conversational support tools, mood and self-tracking platforms, and fully gamified serious games or digital therapeutics with published clinical evidence. Each application was installed and used for a minimum of ten days, covering onboarding flows, core modules/exercises, tracking functions, reminders, guidance systems, and, when accessible, advanced/premium features. Apps were coded along three dimensions frequently discussed in the digital health interaction literature: visual style, functional architecture, and communication tone (37, 38). Inter-rater agreement was assessed using Cohen’s κ, and disagreements were resolved through discussion.

Findings from this analysis are reported in the Results section and were used to inform the design choices described in the remainder of the manuscript (**Table 1**).

**Table 1:** Summarises the reviewed applications, their core clinical engines, engagement and guidance mechanisms, the role of evidence (where applicable), and the specific design/functional elements translated into My Cosmos.

Application	Selection stream	Core clinical engine	Engagement mechanisms	Personalisation/guidance	Published evidence	Elements integrated into My Cosmos
Headspace	App-store benchmark (UX/UI)	Mindfulness psychoeduca tion; guided meditation	Daily routines; brief audio sessions	User-selected goals/content paths	Not assessed (bench mark only)	Minimalist visual style; brief grounding/breathing practices
Balance	App-store benchmark (UX/UI)	Mindfulness; stress reduction	Daily check-ins; progressive programmes	Adaptive content via self-reports	Not assessed (bench mark only)	Adaptive pacing; daily self-reflection prompts
Daylio	App-store benchmark (UX/UI)	Mood/activity self-monitoring	Streaks; visual statistics	Custom reminders	Not assessed (bench mark only)	Simplified mood tracking and visual feedback
Breeze	App-store benchmark (UX/UI)	CBT-informed self-reflection	Exercises; quizzes	Rule-based feedback	Not assessed (bench mark only)	Guided identification of emotional triggers
Wysa	App-store benchmark (UX/UI)	CBT-informed conversational support	Chat-based micro-interventions	Automated conversational guidance	Not assessed (bench mark only)	Structured empathic virtual coach (tone, micro-guidance patterns)
Deprexis	Evidence-supported intervention	Modular CBT; behavioural activation	Dialogic text modules	Branching logic	39; 40; 41	Sequential therapeutic modules; adaptive progression
MoodGYM	Evidence-supported intervention	CBT for depression/anxiety	Quizzes; psychoeducational tasks	Mostly static pathways	42	Interactive psychoeducat ion and exercises
MumMoodB ooster	Evidence-supported intervention	CBT for postnatal depression	Weekly sessions; SMS encouragement	Minimal clinician guidance	43	Scheduled modules; motivational messaging patterns
Moodbuster (ULTEMAT framework)	Evidence-supported adjacent platform/fram ework (EMA-enabled)	EMA/EMI framework supporting mood/anxie ty self-management	Progress charts; homework; self-monitoring	Therapist-linked options (varies by implementation)	44	EMA/EMI monitoring logic; progress visualisations
SPARX	Evidence-supported serious game	CBT-based serious game for depression	Narrative quests; avatars	Difficulty scaling	30	Narrative progression; CBT skill rehearsal via role-play framing
SuperBetter	Evidence-supported intervention	CBT-informed positive psychology	Quests; points; power-ups	User-defined challenges	33	Daily challenges and motivational feedback
smartCAT	Evidence-supported intervention	CBT for childhood anxiety (ecological momentary intervention)	Gamified exercises	Protocol-driven tailoring	45	Gamified CBT skills practice patterns
EndeavorRx (AKL-T01)	Evidence-supported adjacent DTx benchmark	Cognitive training for attention (ADHD)	Adaptive gameplay	Real-time difficulty adjustment	46	Adaptive challenge calibration concepts
Recovery Record	Clinician-link benchmark (not mapped as evidence-supported here)	CBT-informed eating disorder self-monitoring	Self-monitoring; rewards	Clinician dashboard (where available)	Not assessed (bench mark only)	Bidirectional patient– clinician monitoring logic (dashboard inspiration)
reSET	Regulatory/DTx benchmark (not mapped as evidence-supported here)	Digital CBT for substance use disorders	Structured CBT modules; contingency-management tasks	Clinician monitoring/feedback (implementati on-dependent)	8	Structured module logic; clinician-linked monitoring concepts
emoTIC	Serious-game benchmark (not mapped as evidence-supported here)	Socio-emotional skills training (CBT-informed)	Game challenges; narrative progression	Protocol-driven, age-targeted	Not assessed (bench mark only)	Serious-game mechanics for emotional skills acquisition (mechanic-level inspiration)
PTSD Coach	Evidence-informed benchmark (not mapped as evidence-supported here)	CBT-based psychoeduca tion; coping tools	Self-assessment; audio tools	User-controlled pathways	Not assessed (bench mark only)	Symptom tracking and coping-tool pattern ideas
Help ID	Benchmark (not mapped as evidence-supported here)	CBT + ACT + systemic components	Interactive exercises; reminders	Individualised programme	Not assessed (bench mark only)	Modular structure; blended-care positioning concepts
Velibra	Benchmark (not mapped as evidence-supported here)	Transdiagno stic CBT for anxiety disorders	Structured online modules; self-reflection	Algorithm-driven tailoring (implementati on-dependent)	Not assessed (bench mark only)	Transdiagnost ic CBT logic; symptom-driven sequencing concepts

### 2.2 Clinical protocol

The clinical protocol underlying My Cosmos integrates established Cognitive Behavioural Therapy (CBT) frameworks with selected third-wave and metacognitive approaches within a transdiagnostic, modular structure.

Within the predefined six-module pathway, first-wave CBT strategies (behavioural activation/approach behaviour, cognitive restructuring, and problem solving) constitute the core active ingredients. Third-wave and metacognitive elements (e.g., brief mindfulness-based practices, ACT-informed values prompts, and rumination monitoring/metacognitive exercises) are integrated as adjunctive micro-skills intended to strengthen self-regulation and reduce perseverative thinking, rather than as standalone alternative modules. Future efficacy testing will evaluate the integrated protocol as specified; subsequent work may examine the incremental contribution of adjunctive elements.

The protocol targets cognitive, behavioural, physiological, and interpersonal processes shared across anxiety and depressive disorders and is designed for self-guided delivery in adults with mild-to-moderate symptom severity.

Therapeutic content is delivered through an experiential, role-based format in which help-seekers engage with CBT techniques by assuming the role of a psychotherapist working with simulated patients. This design supports structured skills rehearsal (e.g., case formulation, cognitive and behavioural interventions) and facilitates the transfer of therapeutic strategies to real-world contexts.

Core pathways are predefined to support standardisation and evaluation, while the modular architecture allows optional clinical involvement within stepped-care or blended-care models. When clinicians tailor pathways by including or excluding specific modules, individual techniques remain evidence-based, whereas the personalised configuration reflects clinical judgement beyond the validated protocol framework.

Across tracks, progression follows a structured weekly sequence combining interactive in-app activities and real-world homework, with engagement mechanisms embedded within the therapeutic flow to support adherence without altering core clinical content.

#### 2.2.1 Baseline triage and safety gateway

Immediately after registration, the platform runs an automated eligibility and safety screen using validated self-report measures: the Patient Health Questionnaire-9 (PHQ-9) for depressive symptoms (47) and the Generalized Anxiety Disorder-7 (GAD-7) for anxiety symptoms (48). Eligibility thresholds are set to target mild-to-moderate symptom severity. Specifically, users with PHQ-9 ≥ 15 and/or GAD-7 ≥ 15 are redirected to higher- intensity/specialist services and do not proceed in the self-guided pathway.

In the current prototype, this redirection is implemented as an automated “hard-stop” gate: users who exceed eligibility thresholds cannot proceed to the self-guided pathway and are shown a brief explanation that the programme is intended for mild-to-moderate symptom severity. The interface then provides signposting to higher-intensity options (e.g., primary care and local mental health services) and one-tap access to crisis resources. In clinician-supported deployments or research contexts, the same rule can generate a risk/eligibility flag in the clinician dashboard to support timely follow-up according to local governance procedures.

Track assignment follows a rules-based logic using standard cut-offs:

- Anxiety track: GAD-7 ≥ 10 and PHQ-9 < 10
- Depression track: PHQ-9 ≥ 10 and GAD-7 < 10
- Mixed track: PHQ-9 ≥ 10 and GAD-7 ≥ 10

Health-related quality of life is assessed with the SF-36 (49). SF-36 domain scores are used to tailor framing and content density within the assigned track (e.g., pacing, perceived burden).

Safety gateway. Any endorsement of suicidal ideation on PHQ-9 item 9 (score > 0) triggers immediate exclusion from the self-guided pathway, with tailored safety messaging and direct links to crisis resources. This conservative approach reflects standard governance considerations for low- intensity digital CBT delivery at scale and emphasises automated screening and clear escalation routes (25, 37).

#### 2.2.2 Architecture of the six-module pathway

All tracks share six sequential modules. Each module is subdivided into Rehabilitation Objects (ROs), brief interactive learning units coupling psychoeducation with practice tasks, short AR scenes, or mini serious games.

The module order is designed to move from shared psychoeducation and a CBT rationale (Module I) to early behavioural self-regulation skills that can generate rapid “early wins” and build self-efficacy (Module II), followed by cognitive change and problem-solving strategies (Module III). The pathway then targets transdiagnostic maintaining processes such as perseverative thinking and avoidance (Module IV), before addressing interpersonal maintenance factors and assertive communication (Module V), and ending with consolidation and relapse prevention (Module VI). This sequencing was primarily theory-driven (CBT case formulation and graded skills acquisition) and was refined through co-design feedback emphasising clarity, progressive challenge, and early improvements to support motivation.

Rationale and rules for mastery-based progression. Homework is assigned at the end of each week; subsequent modules remain locked until (i) homework is submitted and (ii) embedded quizzes are completed at or above a pre-defined pass threshold. Mastery gating was selected to promote spaced practice, skills rehearsal, and accountability, and to ensure that key skills are rehearsed before new content is introduced. These decision rules were initially set pragmatically during prototyping and iteratively refined based on co-design feedback about goal clarity and the motivational value of “unlocking” subsequent content; parameters will be further optimised during beta testing (**Table 2**).

**Table 2:** Core module configuration across tracks.

Module	Anxiety track	Depression track	Mixed track
I	Psychoeducation on anxiety physiology and CBT rationale	Psychoeducation on depressive cycles and CBT rationale	Dual psychoeducation focusing on emotional dysregulation
II	Behavioural techniques: slow breathing, muscle relaxation, sleep hygiene	Behavioural activation: pleasant/useful activities, energy budgeting	Behavioural activation + relaxation toolbox
III	Cognitive strategies (ABC model, distortions, restructuring)	Same cognitive strategies + mood diary integration	Same cognitive strategies delivered over two weeks
IV	Rumination monitoring and experiential avoidance reduction (MCT- informed)	Rumination monitoring and experiential avoidance reduction	Shared content with track- specific homework emphasis
V	Interpersonal skills: IMS recognition and assertive communication	Same interpersonal skills module	Same interpersonal skills module
VI	Relapse prevention consolidation + relapse signature mapping	Relapse prevention consolidation	Relapse prevention consolidation

#### 2.2.3 Temporal unfolding of each track

The timetable differs slightly in length and weekly focus. “Digital activities” refers to ROs completed in-app; “Real-world homework” describes tasks transferred to daily life (**Tables 3**–**5**).

**Table 3:** Anxiety track (8 weeks).

Week	Module(s) & digital activities	Real-world homework
1	Module I (psychoeducation) & Module II launch	Twice-daily breathing rate log; muscle tension diary
2	Module III-A (ABC modelling, distortion quiz)	Continue week 1 logs; create three ABC sheets
3	Module III-B (restructuring, problem solving)	Two restructuring sheets; two problem-solving tasks
4	Module IV-A (rumination awareness, functional analysis)	Three rumination logs; restructuring review
5	Module IV-B (behavioural approach to avoidance)	Avoidance diary; graded task/exposure schedule
6	Module V-A (IMS recognition with video stimuli)	Identify IMS in three personal episodes
7	Module V-B (assertive dialogue role play)	Practice assertive scripts in two live interactions
8	Module VI (relapse prevention workshop, quiz)	Final self-evaluation; optional module repetition

**Table 4:** Depression track (8 weeks).

Week	Module(s) & digital activities	Real-world homework
1	Module I + Module II launch (BA planner)	Daily mood diary; adherence to activity calendar
2	Module III-A (ABC, distortion quiz)	Maintain diary & planner; three ABC sheets
3	Module III-B (restructuring, problem solving)	Two restructuring + two problem-solving sheets
4	Module IV-A (rumination monitoring)	Functional analysis of three rumination episodes
5	Module IV-B (avoidance alternatives)	Implement two approach behaviours
6	Module V-A (IMS video analysis)	IMS identification; assertive goal setting
7	Module V-B (assertiveness practice)	Execute three assertive communications; self-rate outcomes
8	Module VI (relapse prevention review)	Final self-audit; optional booster planning

**Table 5:** Mixed track (9 weeks).

Week	Module(s) & digital activities	Real-world homework
1	Module I + Module II-A (behavioural activation)	Activity calendar; mood diary
2	Module II-B (relaxation & breathing toolbox)	Breathing + tension logs; continue diary
3	Module III-A (cognition I: ABC modelling, distortion quiz)	Three ABC sheets; distortion quiz
4	Module III-B (cognition II: restructuring, problem solving)	Two restructuring + two problem-solving tasks
5	Module IV-A (rumination focus)	Rumination logs; restructuring review
6	Module IV-B (avoidance focus)	Avoidance diary; approach tasks
7	Module V-A (interpersonal skills I: IMS recognition)	IMS identification in videos and personal events
8	Module V-B (interpersonal skills II: assertiveness)	Assertive communication practice
9	Module VI (relapse prevention)	Skills audit; personalised maintenance plan

#### 2.2.4 Embedded measurement of outcomes

Clinical outcomes are monitored through in-app administration of validated self-report measures: depressive symptoms (PHQ-9; 47), anxiety symptoms (GAD-7; 48), and health-related quality of life (SF-36; 49). Assessments are administered at baseline, at end-of-treatment (Weeks 8–9, track-dependent), and at three-month follow-up. Primary endpoints are changes in PHQ-9 and GAD-7 from baseline to end-of-treatment; secondary endpoints include change in SF-36 scores and maintenance of symptom improvement at follow-up.

In addition to symptom outcomes, engagement and adherence are continuously monitored through passive usage metrics automatically logged by the platform (e.g., module completion rates, number of sessions, total time spent in the app, login frequency, time on task, and homework submission

rates). Such indicators are widely used to characterise engagement in digital mental health interventions and to support implementation monitoring and optimisation (50).

When deployed in blended-care contexts, outcome measures and engagement indicators are visualised within a secure clinician dashboard to support monitoring and timely adherence support. This embedded, measurement-based approach aligns with current reporting and evaluation frameworks for eHealth interventions and with evidence on user experiences and engagement in mental health apps (**Table 6**) (38, 51).

**Table 6:** Outcome measures and engagement metrics embedded in My Cosmos.

Measure type	Instrument/metric	Assessment schedule	Purpose
Primary clinical endpoints	PHQ-9; GAD-7	Baseline; end of programme (Week 8–9)	Quantify change in core anxiety and depressive symptoms
Secondary clinical endpoints	SF-36	Baseline; end of programme; 3-month follow-up	Assess change in health-related quality of life
Symptom maintenance	PHQ-9; GAD-7	3-month follow-up	Assess maintenance of symptom improvement
Passive engagement metrics	Sessions completed; module completion rate; total minutes of app use; time on task; login frequency; % homework submitted; in-app mood ratings	Continuous (automatically logged)	Quantify engagement, adherence, and risk of dropout
Analytic use (blended-care contexts)	Clinician dashboard visualisation; adherence alerts	Continuous	Support monitoring and timely adherence support

#### 2.2.5 Theoretical underpinnings and therapeutic processes

The clinical content of My Cosmos is grounded in evidence-based CBT principles, integrated with selected components of third-wave and transdiagnostic models. Therapeutic strategies were operationalised through structured modules and interactive exercises that target established mechanisms relevant to both depressive and anxiety symptoms (3, 52, 53).

Physiological regulation strategies—such as slow diaphragmatic breathing and progressive muscle relaxation—are employed to reduce autonomic hyperarousal and somatic tension (53, 54). Sleep hygiene and CBT-informed guidance address sleep–mood bidirectionality and support emotion regulation (55, 56).

Behavioural activation targets anhedonia and behavioural withdrawal by increasing engagement in meaningful and reinforcing activities, with personalised planning and pacing tools integrated into the platform (57, 58). Cognitive strategies aim to modify maladaptive appraisals and reduce cognitive distortions through ABC worksheets, thought records, and restructuring exercises (52, 59). Problem-solving techniques support adaptive coping by promoting systematic analysis of stressors and the implementation of actionable solutions, consistent with standard CBT skill repertoires (52).

Rumination and worry are addressed through monitoring exercises and metacognitive techniques designed to reduce the cognitive attentional syndrome, a transdiagnostic driver of emotional distress (13, 60, 61). Users are prompted to identify avoidance patterns and engage in graded approach behaviours, consistent with exposure principles and long-term evidence for CBT-based interventions across anxiety-related disorders (62).

Interpersonal processes are targeted through an Interpersonal Motivational Systems (IMS) framework, which links distress to motivational states and interpersonal schemas (63). My Cosmos operationalises this through scenario-based tasks that train recognition of interpersonal cues and flexible responding. Assertive communication skills are trained through structured role-plays and scenario-based exercises aimed at enhancing boundary setting, needs expression, and interpersonal effectiveness, consistent with evidence supporting assertiveness training as an evidence-based intervention (64).

Finally, relapse prevention strategies are integrated to consolidate therapeutic gains and support maintenance following programme completion. Users are guided to identify personalised relapse signatures (early warning signs) and to develop proactive coping plans and maintenance routines, consistent with mindfulness-based relapse-prevention approaches within CBT traditions (65).

The range of therapeutic techniques embedded in the platform is summarised in **Table 7**.

**Table 7:** Therapeutic techniques embedded in My Cosmos.

Component	Targeted process	Operationalisation in My Cosmos	Key references
Slow diaphragmatic breathing	Autonomic down- regulation; hyperarousal reduction	Guided breathing practice; breathing logs	53, 54
Progressive muscle relaxation (PMR)	Somatic tension reduction; arousal regulation	PMR exercises and structured routines	53, 54
Sleep hygiene & sleep-focused CBT elements	Sleep stabilisation; emotion regulation support	Sleep routines; behavioural guidance; CBT-I elements	55, 56
Behavioural activation	Counteract anhedonia/withdrawal; increase reinforcement	Activity scheduling; pacing/energy budgeting	57, 58, 66
Cognitive restructuring (ABC, thought records)	Reduce distortions; modify maladaptive appraisals	ABC worksheets; thought records; restructuring exercises	52, 59
Problem-solving therapy	Improve coping and action planning	Problem definition; solution generation; implementation	52
Rumination/worry & metacognitive techniques	Reduce CAS; decrease perseverative thinking	Rumination monitoring; metacognitive exercises	13, 60, 61
Avoidance monitoring & graded exposure	Break avoidance cycle; strengthen approach behaviour	Avoidance monitoring; graded task schedules	62
IMS-informed interpersonal training	Enhance social cognition; interpersonal flexibility	IMS recognition tasks; interactive scenarios	63
Assertive communication training	Boundary setting; needs expression	Role-plays; scenario-based practice	64
Relapse prevention & relapse signature mapping	Maintenance; reduce recurrence	Early warning signs; maintenance planning	65

### 2.3 Design and technological architecture of My Cosmos

The design and engineering of My Cosmos were guided by clinical reliability, scalability, and privacy by design in accordance with GDPR (67). The platform comprises a mobile-first help-seeker application and a secure, web-based dashboard for clinicians and administrators, supported by back-end services implementing pseudonymised data flows and a separation between identity management and clinical data, with role-based access control and audit logging to support accountability.

Interface design follows human-centred and accessibility standards (68, 69), while interoperability with health information systems is supported through established healthcare data standards (70). System reliability is supported through operational monitoring and quality assurance practices, given evidence that technical friction contributes to disengagement and attrition in digital mental health interventions (50).

### 2.4 Stakeholders and user interface ecosystem

Within My Cosmos, three stakeholder groups—help-seekers, mental health professionals, and administrators—interact through complementary interfaces: a help-seeker app, a clinician dashboard, and an administrator console.

The help-seeker app guides individuals from baseline screening through the six-module pathway via brief interactive “Rehabilitation Objects” combining psychoeducation, skills rehearsal, and game-inspired feedback. Progression is supported through mastery-based unlocking, progress visualisation (e.g., PHQ−9/GAD−7 trajectories), and a virtual coach. Optional smartphone-based AR micro-tasks (e.g., behavioural-activation “treasure hunts”) extend selected assignments into daily life to encourage real-world practice without requiring dedicated hardware.

The clinician dashboard is designed for blended-care delivery, presenting longitudinal symptom trends, adherence indicators, and eligibility/safety flags in a workflow-oriented layout to support monitoring and between-session continuity. The administrator console supports governance functions such as role management, consent auditing, and aggregated service monitoring, with audit logging to support accountability.

Interface screenshots are provided in **Supplementary Figures 1**–**S9**.

### 2.5 Usability evaluation

Preliminary usability and acceptance were assessed through participatory workshops with potential end-users (help-seekers) and mental health professionals. Sessions included guided presentations of My Cosmos mock-ups/prototype functions, followed by an anonymous questionnaire capturing demographics and digital familiarity, standardised usability and acceptance measures (System Usability Scale [SUS], Mobile App Usability Questionnaire [MAUQ], and UTAUT2 constructs), and open-ended questions on perceived usefulness, engagement drivers, concerns, and preferred usage mode (71–73). SUS was scored using the standard 0–100 procedure; MAUQ and UTAUT2 scores were aggregated and linearly rescaled to 0–100 for comparability. Open-ended responses underwent inductive content analysis with independent coding and consensus. This paper reports only these early-stage usability/acceptability findings; planned feasibility and effectiveness studies will be reported separately (51, 74, 75).

### 2.6 Results

#### 2.6.1 Findings from the state-of-the-art analysis

Across platforms, three recurrent patterns were observed: minimalist visual layouts and calming colour palettes aimed at reducing perceived clinicality; convergence toward a limited set of functional components, including modular CBT-informed content, self-tracking features, lightweight gamification elements, personalisation mechanisms, and guidance systems; and an empathic, user-centred communication style that deliberately avoids diagnostic jargon, consistent with evidence on destigmatising digital mental health interventions (76).

The analysis also highlighted design and implementation gaps. Mindfulness-oriented applications frequently prioritised relaxation and wellbeing practices while offering comparatively less structured CBT skill training. Conversely, academically developed CBT platforms tended to provide robust therapeutic content but were often less optimised for sustained engagement and mobile-first usability. Fully gamified interventions, while effective within specific populations, often relied on fixed storylines or population-specific targets that may limit transferability to adult transdiagnostic contexts. These observations informed the design goals of My Cosmos, namely to integrate CBT fidelity, wellness-grade accessibility, and clinically aligned gamified mechanics within a single modular platform (see **Table 1**).

### 2.7 Preliminary findings – help-seekers (workshops and focus group)

#### 2.7.1 Sample characteristics

A total of 124 participants completed the post-workshop questionnaire. Most participants were female (83.1%, n = 103), with 16.9% male (n = 21). The mean age was 22.4 years (SD = 5.9; range 20–36). Most respondents were students (95.2%, n = 118), with small proportions employed (2.4%) or student workers (~2%). Educational attainment reflected this profile: 62.1% reported upper secondary education and 37.9% held a bachelor’s degree.

Participants reported high comfort with digital tools (M = 3.91/5, SD = 0.86), while prior use of mental health apps was limited (12.9% yes). Self-rated mood was mid-range (M = 3.12/5, SD = 0.93), consistent with a non-clinical sample interested in self-regulation and prevention.

As this was a predominantly non-clinical student sample evaluating a prototype, usability scores should be interpreted as early indicators of interface clarity and perceived value rather than as evidence of clinical acceptability among symptomatic or service-referred populations (**Table 8**).

**Table 8:** Help-seeker sample characteristics.

Domain	Measure	Summary
N	—	124
Age	Mean (SD), range	22.4 (5.9), 20–36
Gender	Female/Male	83.1% / 16.9%
Education	Upper-secondary/Bachelor	62.1% / 37.9%
Occupation	Students/Employed/Student-workers	95.2% / 2.4%/~2%
Digital comfort (1–5)	Mean (SD)	3.91 (0.86)
Prior mental-health app use	Yes / No	12.9% / 87.1%
Mood (1–5)	Mean (SD)	3.12 (0.93)

#### 2.7.2 Quantitative results: usability and acceptance

Descriptive analyses indicated high perceived usability and positive acceptance of the My Cosmos prototype. The mean System Usability Scale (SUS) score was 78.69/100, which is typically interpreted as good usability (Brooke, 1996; 77). The MAUQ mean score was

5.36/7 (rescaled: 72.71/100), suggesting high usability and satisfaction in an mHealth context (72).

UTAUT2 subscales showed high ease of use (Effort Expectancy 4.19/5; rescaled: 79.84/100) alongside moderate expectations of benefit and intention to use (Performance Expectancy 3.63/5; 65.73/100; Behavioural Intention 3.61/5; 65.32/100) (73). Facilitating Conditions (70.36/100) and Social Influence (67.14/100) suggested a moderately supportive usage context, with overall acceptance reflected in the UTAUT2 total (4.05/5; 76.21/100) (**Table 9**).

**Table 9:** Help-seeker usability and acceptance (rescaled 0–100).

Instrument	Mean (0–100)	Interpretation
SUS	78.69	Above average usability
MAUQ	72.71	High usability & satisfaction
UTAUT2 total	76.21	Overall positive acceptance

#### 2.7.3 Qualitative findings

Open-ended responses converged on four themes: perceived usefulness, motivational factors, concerns/barriers, and preferred usage mode. Participants described My Cosmos as a tool to support emotional self-regulation during periods of overload and as a structured space for reflection and self-monitoring outside acute episodes. Many referred explicitly to situations involving anxiety and stress (e.g., “during situations of strong anxiety,” “for exam-related anxiety,” “periods of intense stress”) and highlighted its potential for “monitoring mood,” “tracking progress,” and “automonitoring.” Some also framed the app as complementary to psychotherapy, particularly “during therapy” or when they “do not have the possibility to speak with the therapist.”

Engagement was linked to clear feedback, visible progress tracking, and reminders calibrated to avoid intrusiveness. Participants frequently emphasized the importance of observing improvements (e.g., “seeing progress,” “seeing improvements,” “concrete results”). Continuous feedback and therapist involvement were described as motivating factors, including “continuous feedback” and use “with the supervision of a therapist.” Elements such as “missions,” “daily goals,” and interactive activities were also mentioned as potentially increasing engagement.

Concerns primarily focused on privacy and data security, including fears that “my data might be disclosed,” that “someone other than the therapist could access my data,” or doubts about data protection procedures. A second recurrent concern involved the possible loss of human interaction, with references to “the lack of a therapeutic relationship” and “loss of direct contact with the professional.” Some participants also expressed uncertainty regarding reliability (e.g., “not 100 percent reliable”) or potential over-reliance on digital tools.

Most participants preferred therapist-guided use, frequently indicating a preference for “supervision of a therapist,” while a smaller proportion preferred autonomous use or a flexible combination of both modalities.

#### 2.7.4 Integrative synthesis

Taken together, these quantitative and qualitative findings suggest that My Cosmos is perceived as usable and acceptable among potential users, while highlighting actionable design requirements related to engagement (feedback, progress visibility, calibrated reminders) and trust (transparent data protection communication and positioning as a complement to human care). These insights informed refinements to onboarding, transparency messaging, and blended-care integration.

### 2.8 Preliminary findings – mental health professionals (workshops and focus group)

#### 2.8.1 Sample characteristics

Nineteen mental health professionals completed the post-workshop questionnaire. Most were female (78.9%, n = 15), with 21.1% male (n = 4). Age ranged 26–59 (mean 31.84, SD 8.12). Participants included psychologists (63.2%), psychotherapists in training (26.3%), and psychiatrists (10.5%). Education was high: 63.2% held a master’s degree, 26.3% completed postgraduate clinical specialization, and 10.5% were enrolled in advanced clinical training. Digital comfort in practice was moderate (M = 3.18/5); prior recommendation/use of mental health apps was limited (21.1%) (**Table 10**).

**Table 10:** Professional sample characteristics.

Domain	Measure	Summary
N	—	19
Age	Mean (SD), range	31.84 (8.12), 26–59
Gender	Female/Male	78.9%/21.1%
Role	Psychologists/Trainees/ Psychiatrists	63.2%/26.3%/10.5%
Digital comfort (1–5)	Mean	3.18
Prior app recommendation	Yes/No	21.1%/78.9%

#### 2.8.2 Quantitative results: usability and acceptance

Professionals reported more cautious evaluations than help-seekers, consistent with concerns about workflow fit, risk management, and clinical responsibility. SUS averaged 54.61/100, indicating

moderate perceived usability (71, 77). We interpret these lower professional SUS/MAUQ ratings as primarily reflecting workflow fit, governance, and clinical accountability requirements (e.g., documentation burden, risk management, and integration into existing service processes) rather than intrinsic end-user usability deficits alone. MAUQ was 50.48/100, suggesting intermediate usability/satisfaction (72). UTAUT2 indicated higher perceived ease of use than perceived benefit: Effort Expectancy was 67.09/100, while Performance Expectancy was 51.25/100 and Behavioural Intention was 46.00/100 (73).

Facilitating Conditions (56.50/100) and Social Influence (59.21/100) reflected moderate contextual support (**Table 11**).

**Table 11:** Professional usability and acceptance (rescaled 0–100).

Instrument	Mean (0–100)	Interpretation
SUS	54.61	Moderate usability
MAUQ	50.48	Intermediate usability/satisfaction
UTAUT2 total	55.48	Cautiously positive acceptance

#### 2.8.3 Qualitative findings

Professional feedback clustered around perceived clinical usefulness, implementation barriers, expected patient reactions, and considerations regarding alliance and privacy.

Clinicians primarily described My Cosmos as an adjunctive tool to support homework and continuity between sessions. Several referred to helping patients “work outside sessions,” “support homework,” and “create continuity in therapeutic work, both in session and at home.” Monitoring functions were also highlighted (e.g., “monitoring the patient,” “feedback on the course of therapy”). Some noted its potential to increase engagement, particularly through its “ludic component” and capacity to “engage patients in homework.”

Perceived barriers mainly concerned patient variability and practical integration. Many clinicians stated that implementation would “depend on the patient,” particularly in relation to age and familiarity with digital tools. Additional concerns involved the possible oversimplification of techniques (“using techniques in a rough way”), the application of standardized processes to heterogeneous clinical presentations, and the practical burden of incorporating the app into routine care (e.g., “too demanding to use daily” or something to “constantly keep up with”). A few clinicians mentioned possible impacts on in-session processes, such as “difficulties in communication in the here and now.”

Expected patient reactions were described as heterogeneous. Clinicians frequently emphasized that responses would “depend on the patient,” with some anticipating curiosity or motivation, while others might be hesitant or skeptical. When asked whether app-based supervision could reduce in-person sessions, most clinicians responded negatively (e.g., “no,” sessions “should remain unchanged”), suggesting that the app is largely viewed as complementary rather than substitutive.

Regarding remote monitoring, most clinicians did not report specific concerns. When concerns were mentioned, they primarily referred to the therapeutic alliance or safeguarding sensitive information.

#### 2.8.4 Integrative synthesis

Overall, professionals viewed My Cosmos as promising for homework adherence, continuity, and monitoring, but emphasized that adoption depends on reducing workflow friction, strengthening onboarding, communicating data protection clearly, and reinforcing a blended-care positioning that protects alliance and avoids over-standardization.

## 3 Discussion

This work describes the theoretical foundations, technical realisation, and planned clinical evaluation of My Cosmos, a gamified, modular dCBT platform designed to expand access to evidence-based psychological care. A recurring tension in digital mental health is that clinically robust content often struggles with engagement, while highly engaging products may dilute evidence-based techniques. My Cosmos addresses this by embedding validated CBT tasks into a calibrated motivational structure (narrative framing, progression, rewards, mastery gating) intended to support adherence without trivializing therapeutic work (22, 24).

Across stakeholder groups, preliminary findings indicate a consistent pattern: potential users emphasized emotional support, intuitiveness, and usefulness for self-regulation; clinicians emphasized continuity, homework support, and monitoring, while requiring strong onboarding and clear governance. Both perspectives converged on a blended-care logic in which digital content complements the therapeutic relationship rather than replacing it.

From an implementation perspective, these observations align with the broader view that outcomes depend not only on therapeutic content, but also on onboarding, monitoring, support intensity, and integration into real-world clinical workflows (25, 78, 79). The platform’s dashboards and safety logic reflect an increasing orientation toward measurement-based care in DMH and support future work on personalized sequencing and stepped- care decision points.

## 4 Limitations and challenges

Several limitations should be acknowledged. First, the triage algorithm relies on baseline self-report thresholds and has not yet been validated against diagnostic interviews, creating the possibility of misclassification in complex cases. More broadly, effect size estimates for CBT interventions vary across meta-analytic syntheses and are sensitive to trial quality and risk of bias, underscoring the importance of rigorous evaluation and transparent reporting in the planned RCT (66). Relatedly, the current safety gateway excludes any help-seeker endorsing suicidal ideation on PHQ−9 item 9 (score > 0). This conservative rule prioritises safety and governance for low-intensity delivery at scale, but it may reduce accessibility and limit generalisability to more symptomatic real-world populations. Future iterations may adopt more nuanced, stepped risk stratification (e.g., human review and clinician-led management pathways) to balance safety with access as the platform moves into service deployments. Future work should compare automated triage to clinician assessment to refine sensitivity and specificity.

Second, although gamification is designed to support engagement, a theoretical risk is that extrinsic rewards may reduce intrinsic motivation for some users (80). The current design attempts to mitigate this risk by linking rewards to therapeutic achievements and skill practice rather than arbitrary activity counts; future iterations may allow users or clinicians to calibrate the intensity of motivational feedback to fit preferences and avoid disengagement among users who dislike game mechanics (81).

Third, generalizability is limited by language (Italian), target symptom severity (mild to moderate), smartphone access, and the convenience nature of early feedback samples. Usability and acceptability in other groups (older adults, adolescents, culturally distinct populations) remain unknown and require targeted testing.

Fourth, the planned RCT cannot be double blinded, and attrition bias remains a concern, particularly in self-guided digital interventions and in app-based trials more broadly (17, 82, 83). Longer-term follow-up beyond three months will also be needed to assess durability of gains and sustained engagement.

Finally, real-world implementation raises organizational and economic issues, including reimbursement, clinician workload, governance of risk management, and sustainability. A future cost-effectiveness analysis and explicit implementation science framework would strengthen translation into routine care.

## Data Availability

The raw data supporting the conclusions of this article will be made available by the authors, without undue reservation.

## Author’s note

My Cosmos was co-conceived by Dr. Sasha Del Vecchio and Prof. Marco Recchioni, combining complementary expertise in clinical psychology and e-learning to design a scalable and engaging digital mental health intervention. Dr. Del Vecchio developed the therapeutic protocol and intervention structure, including the clinical framework and training materials, and contributed to the design of core functionalities to ensure alignment between therapeutic logic and the digital environment. Prof. Recchioni defined the platform’s structural and functional logics, integrating principles of engagement, modular learning, and scalability into its architecture. The technical realisation of the prototype, encompassing software infrastructure, user interface, gamified architecture, and XR components, was implemented by ReMedia, which also contributed to overall system design and coordinated the development process.

## Supplementary material

Supplementary Figure S1 Representative screenshot of the My Cosmos help-seeker app welcome/onboarding interface.

Supplementary Figure S2 Representative screenshot of the baseline assessment and eligibility screening workflow.

Supplementary Figure S3 Representative screenshot of track allocation and module overview within the help-seeker app.

Supplementary Figure S4 Representative screenshot of a Rehabilitation Object delivering psychoeducation and interactive practice.

Supplementary Figure S5 Representative screenshot of behavioural activation/relaxation task content and homework integration.

Supplementary Figure S6 Representative screenshot of progress tracking and symptom-monitoring features in the help-seeker app.

Supplementary Figure S7 Representative screenshot of gamified progression, mastery-based unlocking, and feedback elements.

Supplementary Figure S8 Representative screenshot of the clinician dashboard displaying symptom trends, adherence indicators, and alerts.

Supplementary Figure S9 Representative screenshot of the administrator interface supporting role management, consent auditing, and service monitoring.
