# Supplementary figures and images for: My Cosmos: development, clinical protocol, and preliminary usability of a gamified transdiagnostic digital CBT platform
Source: Front Psychiatry. 2026 Apr 10;17:1792957. doi: 10.3389/fpsyt.2026.1792957 (PMC13106314; doi:10.3389/fpsyt.2026.1792957)

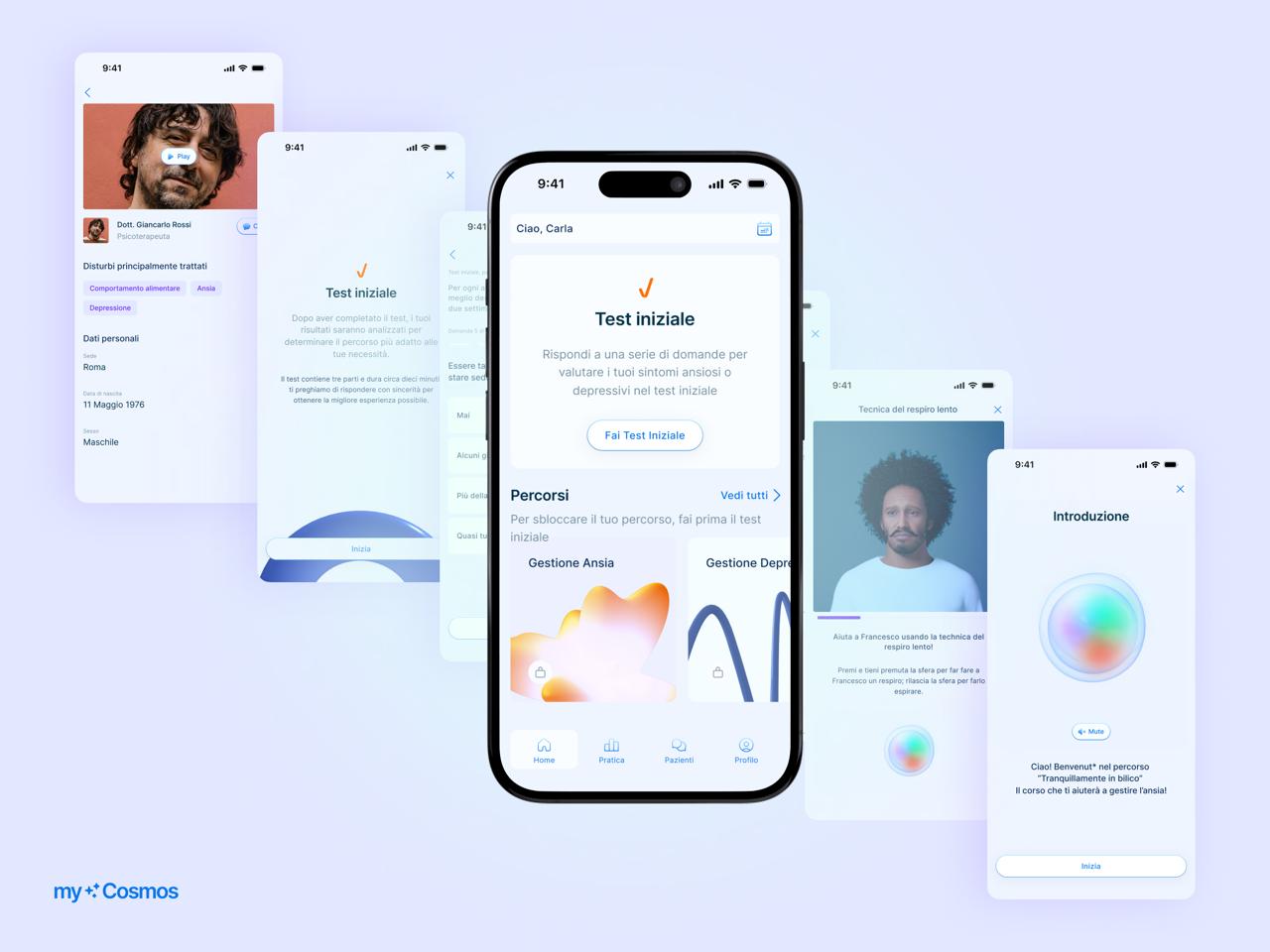

Supplement: Supplementary Figure S1 — Representative screenshot of the My Cosmos help-seeker app welcome/onboarding interface. [file Image1.jpeg]

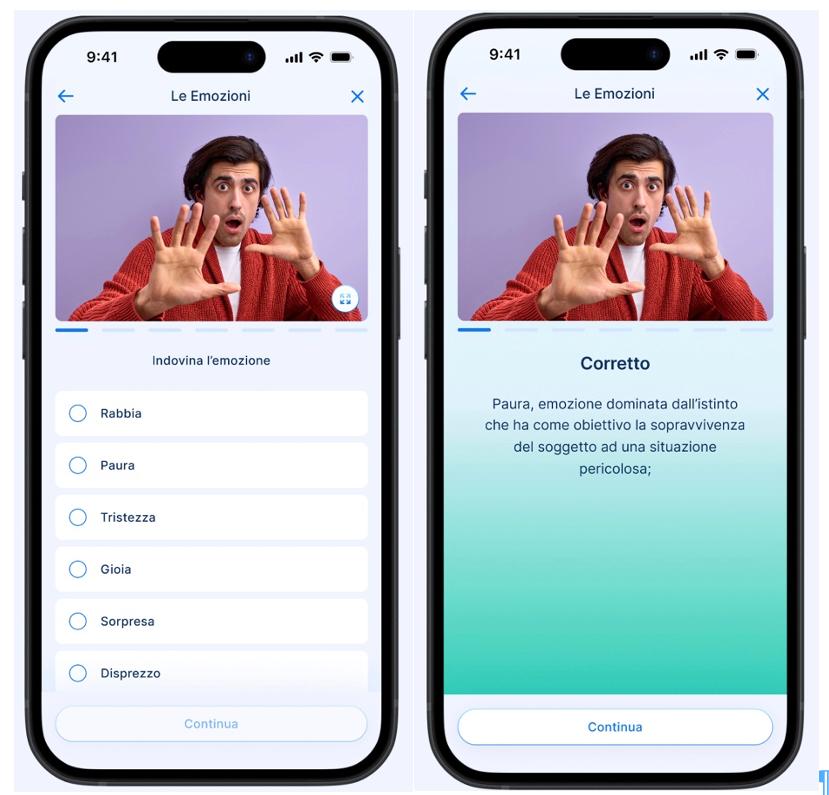

Supplement: Supplementary Figure S2 — Representative screenshot of the baseline assessment and eligibility screening workflow. [file Image2.jpeg]

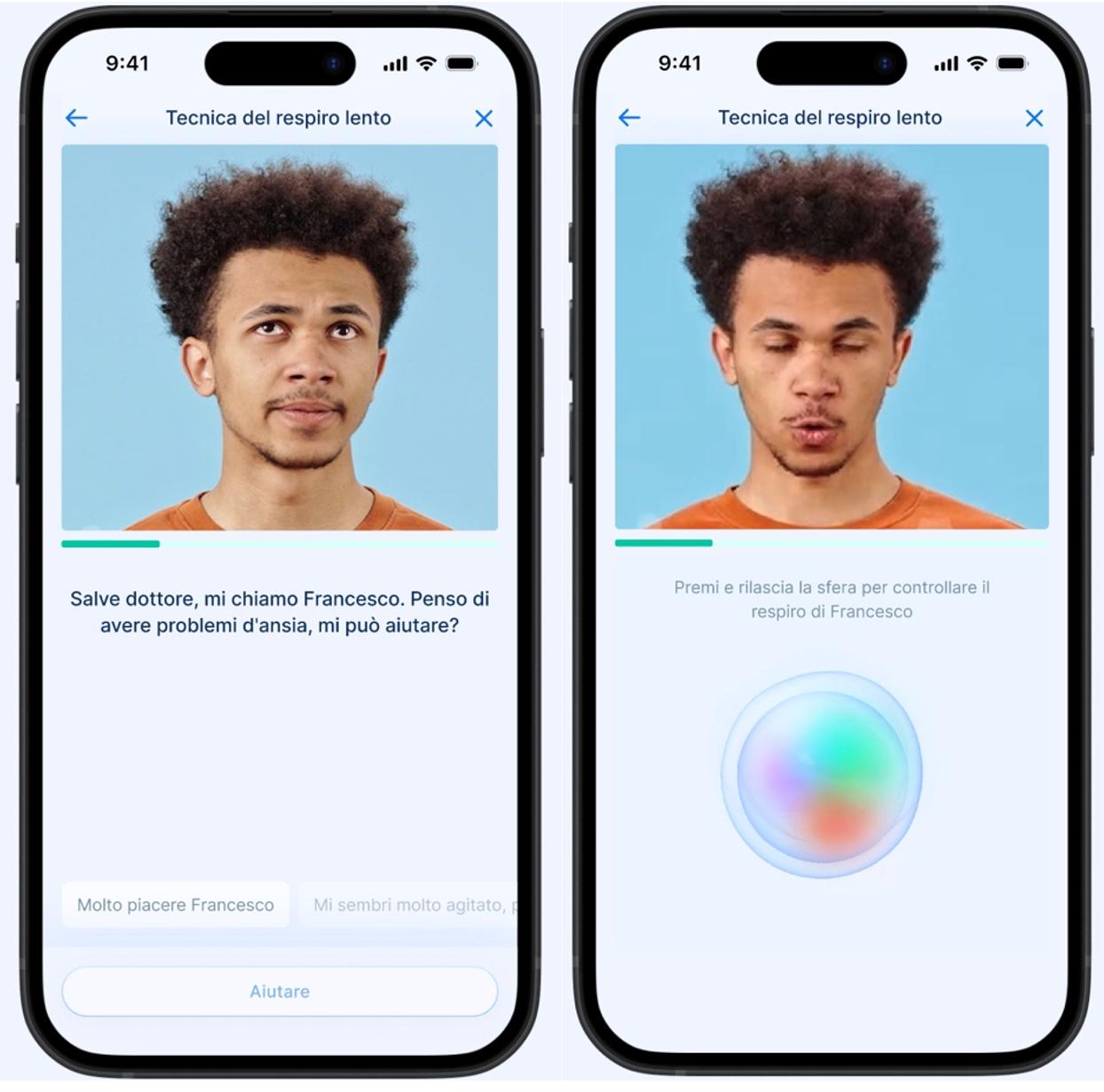

Supplement: Supplementary Figure S3 — Representative screenshot of track allocation and module overview within the help-seeker app. [file Image3.jpeg]

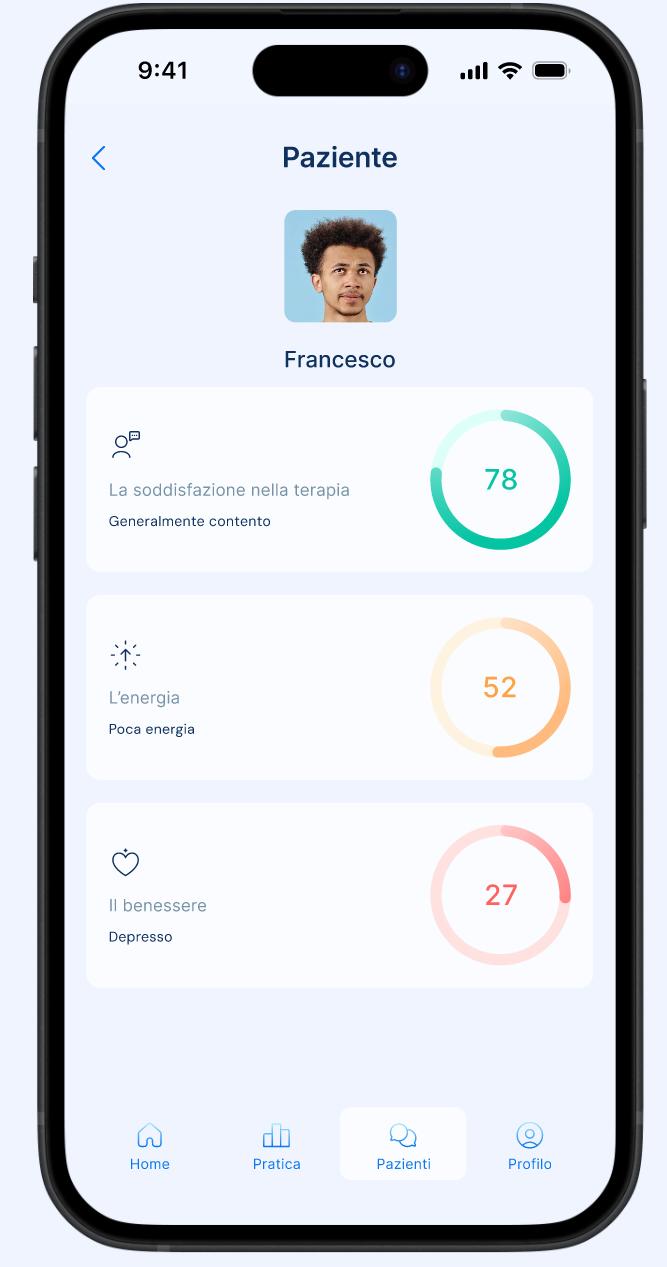

Supplement: Supplementary Figure S4 — Representative screenshot of a Rehabilitation Object delivering psychoeducation and interactive practice. [file Image4.jpeg]

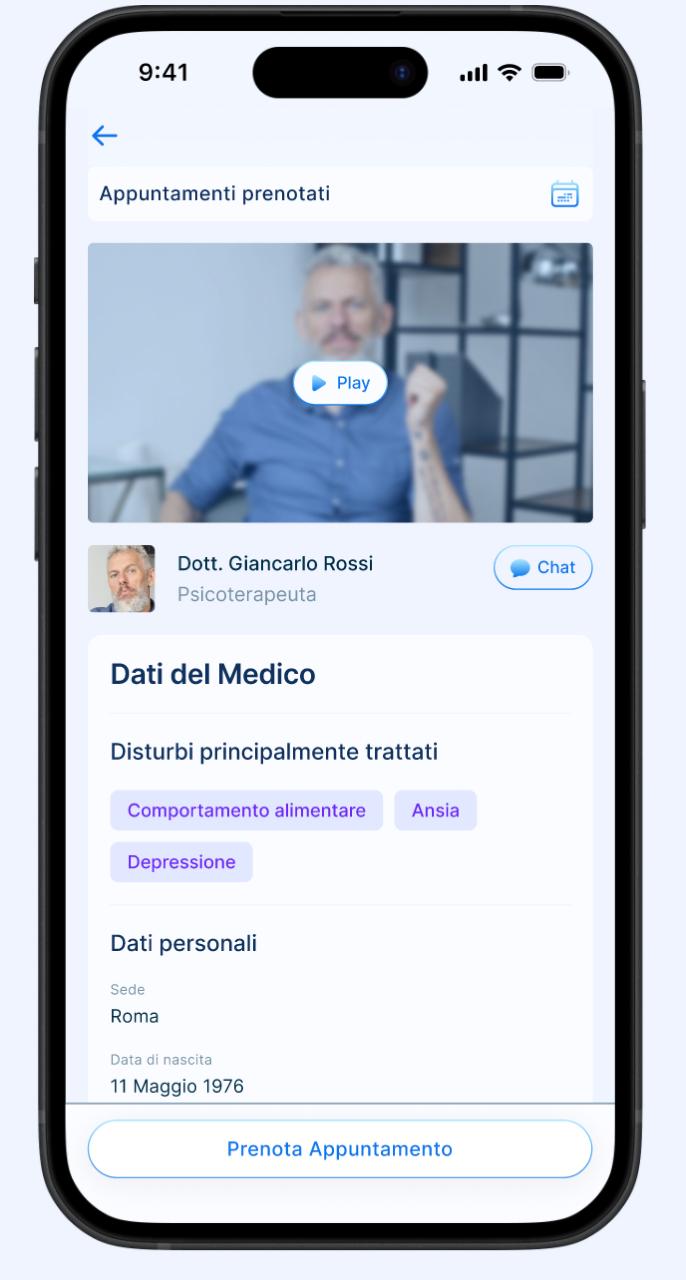

Supplement: Supplementary Figure S5 — Representative screenshot of behavioural activation/relaxation task content and homework integration. [file Image5.jpeg]

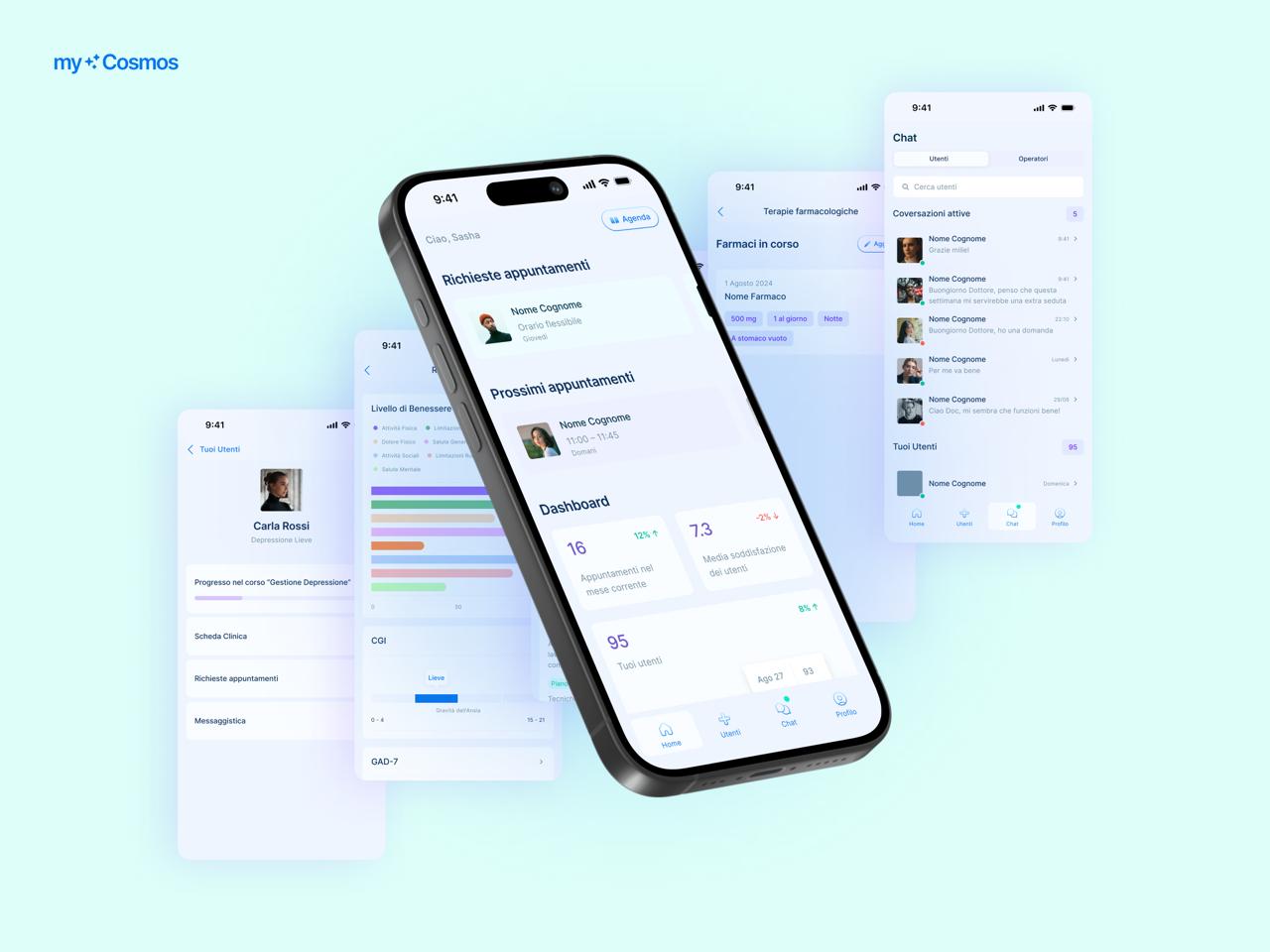

Supplement: Supplementary Figure S6 — Representative screenshot of progress tracking and symptom-monitoring features in the help-seeker app. [file Image6.jpeg]

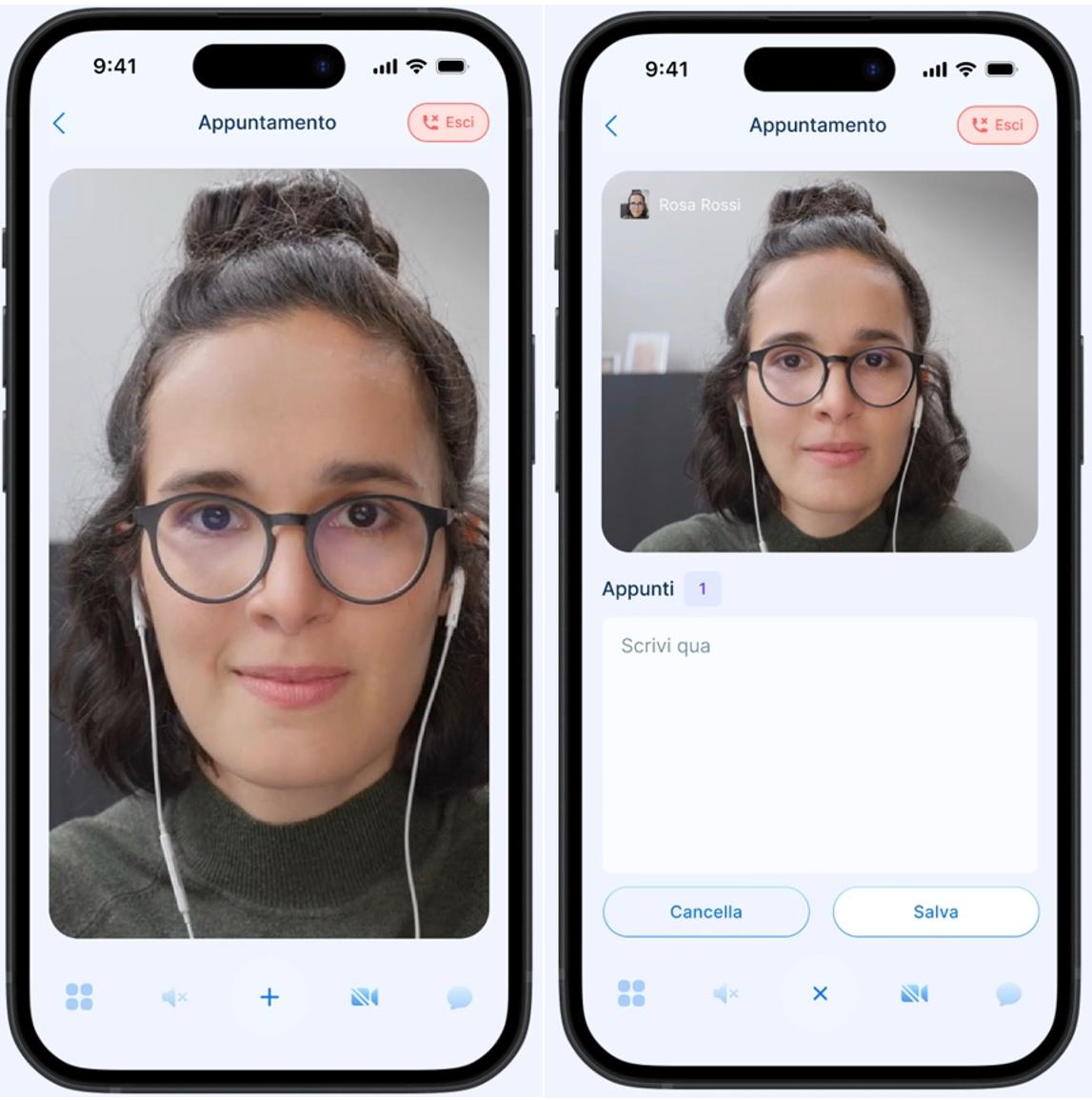

Supplement: Supplementary Figure S7 — Representative screenshot of gamified progression, mastery-based unlocking, and feedback elements. [file Image7.jpeg]

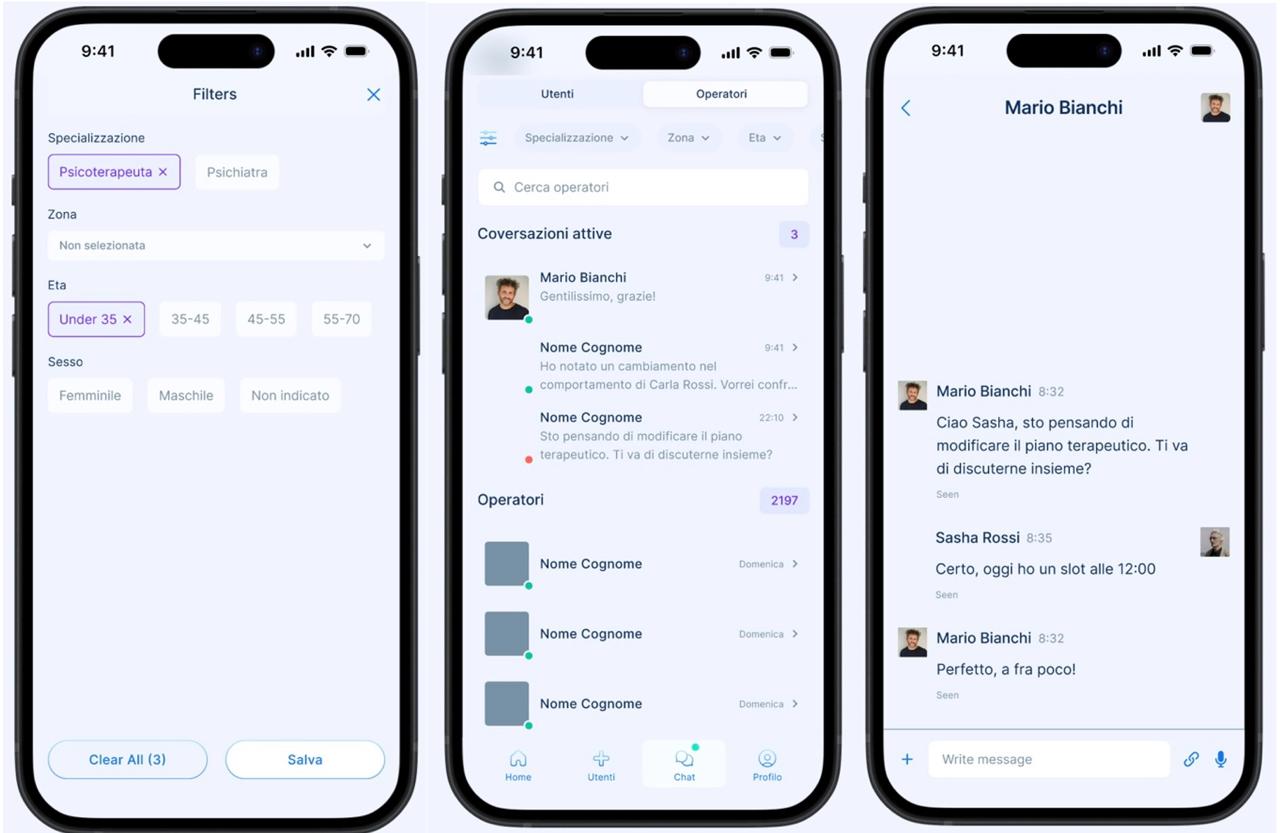

Supplement: Supplementary Figure S8 — Representative screenshot of the clinician dashboard displaying symptom trends, adherence indicators, and alerts. [file Image8.jpeg]

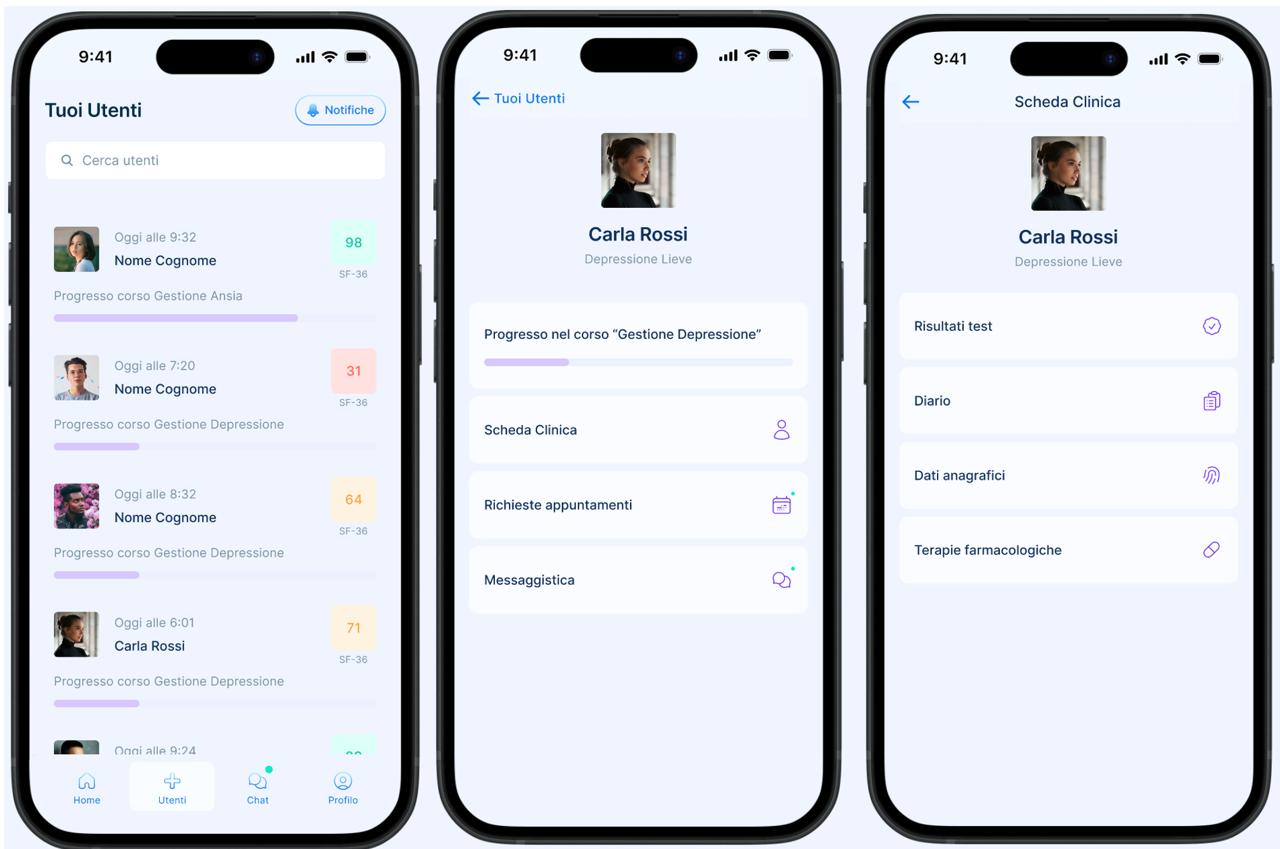

Supplement: Supplementary Figure S9 — Representative screenshot of the administrator interface supporting role management, consent auditing, and service monitoring. [file Image9.jpeg]
